# Calreticulin Fragment 39-272 Promotes B16 Melanoma Malignancy through Myeloid-Derived Suppressor Cells *In Vivo*

**DOI:** 10.3389/fimmu.2017.01306

**Published:** 2017-10-12

**Authors:** Xiao-Yan He, Fang-Yuan Gong, Yong Chen, Zhe Zhou, Zheng Gong, Xiao-Ming Gao

**Affiliations:** ^1^School of Biology and Basic Medical Sciences, Institute of Biology and Medical Sciences, Soochow University, Suzhou, China

**Keywords:** calreticulin, MDSC, melanoma, S100A8/9, TLR4

## Abstract

Calreticulin (CRT), a multifunctional Ca^2+^-binding glycoprotein mainly located in the endoplasmic reticulum, is a tumor-associated antigen that has been shown to play protective roles in angiogenesis suppression and anti-tumor immunity. We previously reported that soluble CRT (sCRT) was functionally similar to heat shock proteins or damage-associated molecular patterns in terms of ability to activate myeloid cells and elicit strong inflammatory cytokine production. In the present study, B16 melanoma cell lines expressing recombinant CRT fragment 39-272 (sCRT/39-272) in secreted form (B16-CRT), or recombinant enhanced green fluorescence protein (rEGFP) (B16-EGFP), were constructed for investigation on the roles of sCRT in tumor development. When s.c. inoculated into C57BL/6 mice, the B16-CRT cells were significantly more aggressive (in terms of solid tumor growth rate) than B16-EGFP controls in a TLR4- and myeloid-derived suppressor cells (MDSC)-dependent manner. The B16-CRT-bearing mice showed increased Gr1^+^ MDSC infiltration in tumor tissues, accelerated proliferation of CD11b^+^Ly6G^+^Ly6C^low^ (G-MDSC) precursors in bone marrow, and higher percentages of G-MDSCs in spleen and blood, which was mirrored by decreased percentage of dendritic cells (DC) in periphery. In *in vitro* studies, recombinant sCRT/39-272 was able to promote migration and survival of tumor-derived MDSCs *via* interaction with TLR4, inhibit MDSC differentiation into DC, and also elicit expression of inflammatory proteins S100A8 and S100A9 which are essential for functional maturation and chemotactic migration of MDSCs. Our data provide solid evidence for CRT as a double-edged sword in tumor development.

## Introduction

Calreticulin (CRT) is a Ca^2+^-binding protein mainly located in the lumen of endoplasmic reticulum of the cell, where it controls intracellular calcium homeostasis and also functions as a molecular chaperon (1). In addition, CRT is also found on the membrane surface as well as other cellular organelles and can be released in soluble form into extracellular space during cell death. This multifunctional protein plays diverse roles in physiological and pathological processes such as cell adhesion, wound healing as well as recognition and elimination of apoptotic cells (2).

Furthermore, CRT is known as a tumor-associated antigen (TAA) for its significantly increased expression in various tumor cells (e.g., melanoma) and also the presence of soluble CRT (sCRT) in body fluids of patients with different types of cancers (3–5). Tumor immunobiological studies uncovered evidence suggesting positive roles for CRT in the control of tumor development. In earlier studies, the N-terminal domain (amino acid residues 1–180) of CRT was named “vasostatin” for ability to inhibit endothelial cell proliferation *in vitro* and angiogenesis *in vivo* (6). More recent work indicated that sCRT augmented adhesion molecule expression on endothelial cells and led to increased T cell infiltration in tumor tissues (7). Cell-surface CRT (ecto-CRT) on apoptotic tumor cells, induced by chemotherapeutic drugs or radiation, functions as an “eat me” signal for activating receptors such as LDL-receptor-related protein of phagocytic cells (8). Since tumor-derived CRT molecules often carry with them tumor-derived antigens (or antigenic peptides thereof), they can effectively elicit tumor antigen-specific adaptive cellular responses *in vivo* (9). Despite the above progress, however, no evidence has been presented showing that downregulation of CRT expression could either serve as a means of tumor escape from the immunological pressure or correlate with increased malignancy. The question whether CRT is a vicious contributor or an important limiting factor to tumor development *in vivo* remains unclear.

CD11b^+^Gr1^+^ myeloid-derived suppressor cells (MDSCs) are a heterologous population of myeloid cells in tumor microenvironment arrested at an immature stage and capable of modulating cellular immunity and promoting tumor development in humans and mice (10, 11). Two major subsets of CD11b^+^ MDSCs have been identified according to their surface expression of Ly6G and Ly6C: G-MDSCs (Ly6G^+^Ly6C^low^, a granulocytic phenotype) and M-MDSCs (Ly6G^−^Ly6C^high^, a monocytic phenotype) (10), with the former much more strongly associated with tumorigenesis than the latter (11). TLR4 is a major activating receptor on the surface of both subsets (12, 13). Based upon our previous finding that a water soluble fragment of CRT covering N-terminal amino acid residues 39–272 (CRT39-272, including the N domain and partial P domain) is capable of activating myeloid cells including dendritic cells (DCs), monocytes, and macrophages though CD14/TLR4 receptor complex (14, 15), we hypothesized that tumor-derived sCRT might be able to promote MDSC generation, proliferation, and/or chemotaxis through TLR4, thereby contributing to tumor malignancy *in vivo*. In the present study, we demonstrate that B16 cells (a mouse melanoma tumor line) expressing recombinant sCRT39-272 exhibit significantly more malignant behavior in mice, which is attributed to the ability of sCRT39-272 to expand and recruit MDSCs in a TLR4 and S100A8/9-dependent manner.

## Materials and Methods

### Cell Lines

The B16 melanoma cell line was purchased from the Cell Bank of the Chinese Academy of Sciences (Shanghai, China) and cultured in Dulbecco’s modified Eagle’s medium (DMEM) supplemented with 10% fetal bovine serum and 100 U/ml penicillin/streptomycin (Invitrogen). B16 melanoma cells were retrovirally transfected with mouse CRT fragment 39–272 cDNA and a leading sequence at the N-terminal (B16-CRT) or with empty vector (B16-EGFP) using LV5-CRT-Puro lentivirus vector. Transfection efficiency was estimated by enhanced green fluorescence protein (EGFP) fluorescence intensity in the transduced cells that were propagated in medium containing 5 µg/ml Puromycin (Sigma, USA). sCRT in supernatant was detected by Western blot analysis.

### Mice

Female C57BL/6 (H-2^b^) mice and TLR4-knockout mice of C57BL/6 background of 6–8 weeks of age were purchased from the Model Animal Research Center of Nanjing University (Nanjing, China). CD45.2 mice on C57BL/6 background and OT-I mice (transgenic for TCR against chicken ovalbumin peptide 257–264) were generously provided by Dr. H Liu (Suzhou University, Suzhou, China). Mouse experimental procedures were performed under specific pathogen-free conditions.

### Cell Adhesion Assay

Cells were seeded at 10^5^ per well into 96-well flat-bottomed plates precoated with laminin (10 µg/ml) or fibrinogen (200 µg/ml) and incubated for 30 min. Unadhered cells were washed out twice using PBS, and the remaining cells stained with 0.1% crystal violet for 5 min and dissolved by 30% acetic acid. The plates were then read in a spectrometer (Biotek, WA, USA) at 590 nm. Cell adhesion rate was calculated using unwashed control wells as 100%.

### Wound Healing Assay

A total of 5 × 10^6^ cells were seeded into six-well plates with three duplicates and scratched with three parallel vertical lines using 10 µl pipettes. The cells were washed three times with PBS and incubated in serum-free DMEM. Cell motility was evaluated by observing, at intervals of 0, 36, and 72 h, the movement of cells into the scratch area under a microscope. The images were recorded and the area of wound gap was measured by Image-J software. Wound closure (%) = [Gap area (*T*_0_ − *T*)/Gap area *T*_0_] × 100% (where *T* is the treatment time and *T*_0_ is the time when the wound was first induced).

### Tumor Inoculation

Groups of mice were s.c. inoculated with viable B16-CRT, or B16-EGFP cells (5 × 10^6^ cells in 100 µl saline) at the left flank. Tumor growth was monitored every 3 days. Solid tumors formed at the site of injections were measured with fine digital calipers and tumor volume was calculated by the following formula: tumor volume = 0.5 × width^2^ × length. Tumor weight was evaluated postscarification.

For tumor metastasis experiments, mice were injected with B16-EGFP or B16-CRT viable cells (1 × 10^6^ cells in 100 µl saline) *via* the tail vein and monitored for up to 4 weeks. At the end of the experiments, mice were sacrificed for their lungs and livers that were fixed in Bouin’s solution and nodules on the organ surface were counted.

### Flow Cytometry Analysis

Single cell suspensions were treated with fluorescence-labeled Abs against mouse CRT, CD11b, Gr-1, Ly6G, Ly6C, or CD11c (either alone or in different combination) on ice for 1 h, followed by washes and resuspension in fixation buffer. The stained cells were then subjected to flow cytometric analysis on Attune NxT (Life Technology). FACS data were analyzed using FlowJo software.

Cells were prepared from spleen, bone marrow, peripheral blood, and tumor tissues. Red blood cells were lysed using ACK Lysis Buffer (Beyotime, China).

### Purification of Ly6G^+^ MDSCs

Single-cell suspensions of splenotes were prepared by mincing mouse spleens on ice and filtering to remove debris and washed twice in PBS before resuspending in RPMI-1640 complete medium.

Tumor tissue was minced on ice and then digested with Collagense Type I (3 mg/ml, Gibco) and DNase I (0.2 mg/ml, Sigma) at 37°C for 2 h. At the end of incubation, preparations were filtered to remove debris and washed twice in PBS before resuspending in RPMI-1640 complete medium.

Single-cell suspensions of splenotes from B16-CRT or B16-EGFP tumor-bearing C57BL/6 mice were sequentially treated with biotinylated antimouse Ly6G^+^ (Miltenyi Biotech) and antibiotin-coated microbeads, the positively labeled cells were then sorted using LS MACS columns according to the manufacturer’s protocol. The purity of G-MDSC populations was over 90%, as determined by flow cytometry, and the viability was higher than 95% as established by exclusion with Trypan blue.

### Suppression Assay of MDSCs

Freshly isolated MDSCs from B16-CRT- or B16-EGFP-bearing mice were incubated for 72 h together with Dye eFluorR 670 (eBioscience, USA)-labeled splenocytes from OT-I mice at a ratio of 1:2 in the presence of 5 µg/ml OVA257–264 peptide (SIINFEKL, Eurogentec, Belgium) in U bottom 96-well plate. Proliferation of Dye eFluorR 670^+^CD8^+^ T cells was analyzed using flow cytometry.

### MDSC Depletion

For *in vivo* MDSC depletion, C57BL/6 mice were i.p. injected with Gemcitabine (LC Laboratories, MA, USA) at 100 mg/kg on days 1, 3, 6, 9, 12, 15, 18, 21, 24, and 27 after B16 tumor inoculation. MDSC depletion efficiency was assessed by flow cytometric analysis on CD11b^+^Gr1^+^ population among the splenocytes.

### BrdU Incorporation

For *in vivo* BrdU incorporation assay, C57BL/6 mice (*n* = 6)-bearing B16 solid tumors for 18 days were i.p. injected with BrdU (10 mg/ml, 200 µl/mouse) and sacrificed 12 or 4 h thereafter for their bone marrow. For *in vitro* BrdU incorporation assay, BrdU (10 µM final concentration) was added directly to bone marrow culture 2 h prior to harvest. The cells were harvested and stained with fluorescence-labeled anti-CD11b, anti-Ly6G and anti-Ly6C, followed by intracellular staining with anti-BrdU-APC, for flow cytometric analysis.

### Adoptive Transfer

Freshly isolated bone marrow cells (2 × 10^7^) from C57BL/6 mice (CD45.1 genotype) were injected into the tail veins of tumor-bearing C57BL/6 mice of the CD45.2 genotype. Five days later, the recipient animals were sacrificed for their bone marrow cells that were subsequently stained with fluorescence-labeled antibodies against CD45.1 and MDSC makers for flow cytometric analysis.

### Migration Assay

Myeloid-derived suppressor cell migration assays were performed in Transwell tissue culture plates, with transwell filters precoated with fibrinogen to prevent cells falling down. MDSCs freshly isolated from spleens of B16 tumor-bearing mice were placed in the chamber on top of that containing sCRT/39-272 or rEGFP. After 3 h incubation, transwell filters were fixed with 4% paraformaldehyde (PFA) in PBS at RT for 30 min, followed by wiping away the remaining cells in the top chamber and subsequent staining of cell nucli with DAPI for observation under a fluorescence microscope (Nikon A1, Japan).

### Immunofluorescence Staining of Tissue Sections

B16-CRT and B16-EGFP solid tumor tissues from C57BL/6 mice were cut into 5-µm frozen sections and fixed in ice-cold acetone for 30 min. Sections were incubated sequentially with anti-Gr1 (Invitrogen) or Alexafluor 647-labeled goat anti-rat IgG (Invitrogen) secondary antibodies, respectively. The stained sections were analyzed by confocal scanning laser microscopy.

### Statistic

All statistical analyses were performed with GraphPad Prism Software version 4. Unpaired Student’s *t*-tests were used to compare differences between the groups. Error bars represent the SEM of the mean. A *p* value of 0.05 was considered statistically significant.

## Results

### Expression of Recombinant sCRT39-272 Enhances Malignancy of B16 Cells

**Figure 1:**
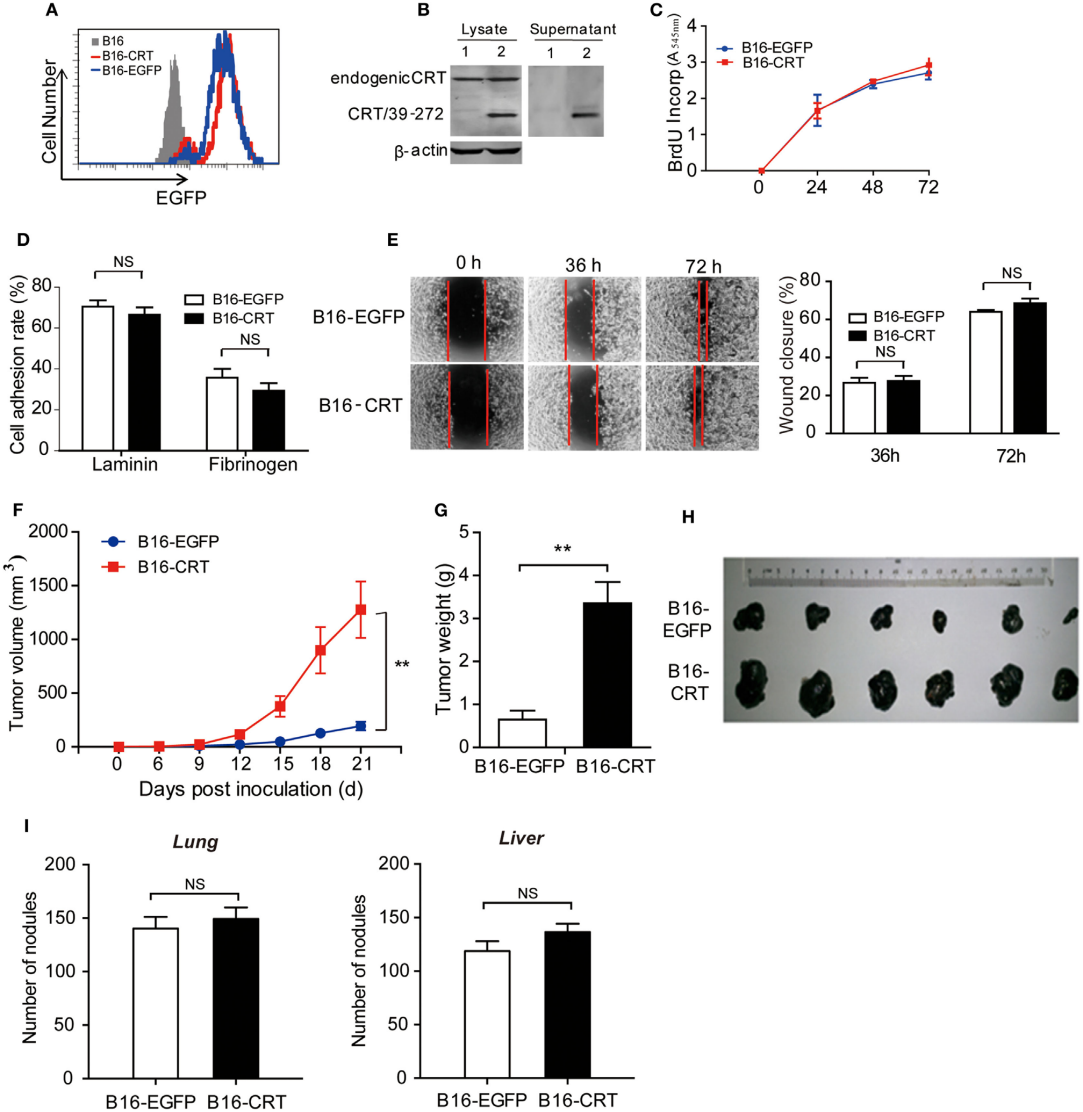
Construction and characterization of the B16-CRT and B16-EGFP cell lines. **(A)** FACS analysis for recombinant enhanced green fluorescence protein (rEGFP) expression in B16, B16-EGFP, and B16-CRT cell lines. **(B)** Total cell lysate and culture supernatant of B16-EGFP (*Lane 1*) and B16-CRT (*Lane 2*) line cells were separated by SDS-PAGE 10% gels, followed by Western blotting using polyclonal rabbit anti-CRT Abs. Beta-actin was included as an internal control. The experiment was repeated three times and a representative blot image is shown. **(C)** Spontaneous proliferation of B16-EGFP and B16-CRT cells in Dulbecco’s modified Eagle’s medium (DMEM) was measured by BrdU incorporation method. **(D)** B16-EGFP and B16-CRT cells were compared in cell adhesion assays on lamnin and fibrinogen surface. **(E)** Representative images of “wound closure” assays on B16-EGFP and B16-CRT cells are displayed. B16-EGFP and B16-CRT cells were grown to confluence in six-well tissue culture plates. Three wounds were scraped in each well with a sterile pipette tip. Mechanically wounded cells were grown in medium and images were taken at 0, 36, and 72 h. The remaining wound area (percentage of residual wound area compared with *t* = 0 h) was measured after 36 and 72 h. Statistics of wound closure results are shown in the right panel. **(F–H)** Groups of female C57BL/6 mice (*n* = 6) were s.c. injected with B16-EGFP or B16-CRT cells (5 × 10^6^/100 μl/mouse). Diameter of solid tumors at the sites of injection was evaluated every 3 days thereafter using a vernier caliper for 21 days. Tumor volume was estimated by the formular of 0.5 × width^2^ × length. Tumor growth curves are shown in panel **(F)**. The mice were sacrificed on day 21 for their solid tumors [shown in panel **(H)**] that were weighed **(G)** and used in subsequent studies. **(I)** Female C57BL/6 mice were i.v. injected with B16-EGFP or B16-CRT cells (10^6^/100 μl/mouse) and sacrificed 28 days later for their lungs and livers. Tumor nodules on the organ surface were counted under a dissecting microscope. **p < 0.01. NS, not significant. The experiment was repeated for four times, and representative results are shown.

Although CRT is constitutively expressed in cells and can be released in soluble form during cell death, studies on the contribution of tumor-derived sCRT in tumor formation and development face many obstacles. In the present study, we constructed a murine B16 melanoma cell line expressing recombinant sCRT39-272 (B16-CRT), with recombinant EGFP (rEGFP) as a coexpression marker. B16 cells expressing rEGFP (B16-EGFP) alone were included as control (Figures 1A,B). There was virtually no difference between these two cell lines in terms of spontaneous proliferation, adhesion to lamnin- or fibrinogen-coated surface, and Transwell migration (Figures 1C–E), indicating that sCRT39-272 expression *per se* was unlikely to have direct effects on B16 malignancy. When s.c. inoculated into C57BL/6 mice (5 × 10^6^/mouse), however, the B16-CRT line appeared to be much more aggressive than the B16-EGFP control. Solid tumor formation at the sites of injection was observable 12 days postinoculation of the B16-CRT cells, 3 days earlier than that of the B16-EGFP control (Figure 1F). By day 21 after inoculation, average volume and weight of the B16-CRT solid tumors were by far more than that of the B16-EGFP controls (Figures 1G,H). It should also be noted that, when B16-CRT and B16-EGFP cells were i.v. injected into C57BL/6 mice (10^6^/mouse), there was no significant difference between the two groups in terms of size and number of solid tumor nodules on the surface of their lungs and livers 4 weeks postinoculation (Figure 1I), suggesting that the expression of sCRT39-272 did not render B16 cells more metastatic.

### Increased MDSC Infiltration in B16-CRT Solid Tumor Tissues

**Figure 2:**
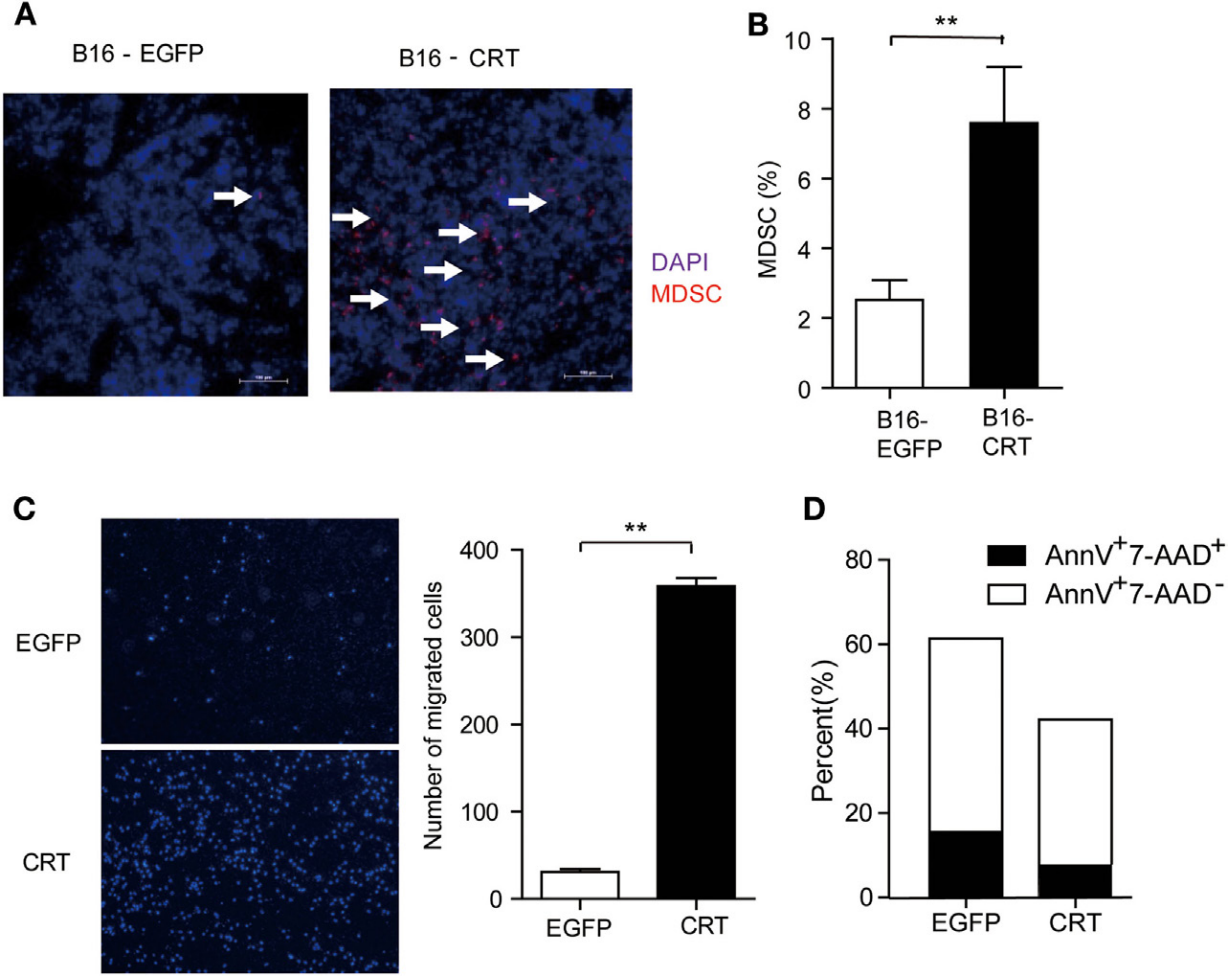
Multiple impacts of sCRT/39-272 on myeloid derived suppressor cells (MDSCs). **(A)** Groups of female C57BL/6 mice (*n* = 6) were s.c. injected with B16-EGFP or B16-CRT cells (5 × 10^6^/100 μl/mouse) and sacrificed 21 days postinoculation for their solid tumors. Tumor tissues were made into 5 μM frozen section for immune-fluorescence staining using Alexafluor 647-labeled anti-Gr1 Abs (red) and DAPI (blue). Images were acquired by laser-scanning confocal microscopy. Arrows indicate Gr1^+^ MDSCs. **(B)** B16-CRT and B16-EGFP tumor tissues were cut into small pieces and digested with collagenase and DNase digestion. Single cells were filtered through 40-µm nylon mesh after further mechanically dissociated with a 10-ml pipette and followed by staining with fluorescence labeled anti-CD11b and anti-Gr1 Abs. Percentage of Gr1-positive MDSCs among the tumor cells was analyzed by FACS. **(C)** For *in vitro* migration assays, MDSCs, purified from the spleens of tumor-bearing mice using biotin labeled anti-Ly6G-coupled microbeads, were placed in the top chamber of a transwell, recombinant sCRT/39-272 or recombinant enhanced green fluorescence protein (rEGFP) was placed in the bottom chamber. After 3 h, transwell filters were fixed with 4% paraformaldehyde for 30 min, followed by wiping away the remaining cells in the top chamber and then DAPI staining, cells was visualized by fluorescence microscope. Representative micrographs are shown on the left and statistic result of 6 different vision cells on the right. **(D)** MDSCs purified from tumor-bearing mice were incubated with sCRT/39-272 or rEGFP for 20 h, followed stained with fluorescence-labeled Annexin-V and 7-AAD and detected by FACS. The results were replicated for three times with consistent trend, representative results of the three repeating experiments are shown. **p < 0.01.

Given that tumor cell-derived sCRT39-272 was unable to directly promote B16 cell proliferation (Figure 1C), its malignancy enhancing effect has to be explained by alternative mechanisms such as proangiogenesis or immunosuppression. Contradictory reports on the antiangiogenisis roles of the CRT N domain have been documented (6, 16). In our hands, recombinant sCRT39-272 was unable to exert any measurable suppression or promotion effect on the growth of blood vessel endothelial cells *in vivo* (data not shown). Consistent with our notion that sCRT39-272 might be able to facilitate the differentiation/generation of MDSCs through CD14/TLR4 signaling, the number of Gr1^+^ cells in tissue sections of B16-CRT solid tumors (harvested from C57BL/6 mice 21 days postinoculation) was substantially higher than that of the B16-EGFP group (Figure 2A), which is confirmed by flow cytometric analysis for CD11b^+^Gr-1^+^ cells among the disseminated cells from the solid tumor tissues (Figure 2B).

The increased presence of CD11b^+^Gr1^+^ MDSCs in B16-CRT solid tumors could be the results of selective chemotaxis and/or *de novo* expansion. The MDSC chemotaxis potential of sCRT39-272 was confirmed by Transwell assays in which sCRT39-272 was significantly more effective than rEGFP in inducing MDSC migration (Figure 2C). Additionally, sCRT39-272 was also able to block their apoptosis *in vitro* (Figure 2D). These results indicate that sCRT/39-272 has multiple impacts on the survival and chemotaxis of MDSCs.

### Increased Expansion of G-MDSCs in B16-CRT-Bearing Mice

**Figure 3:**
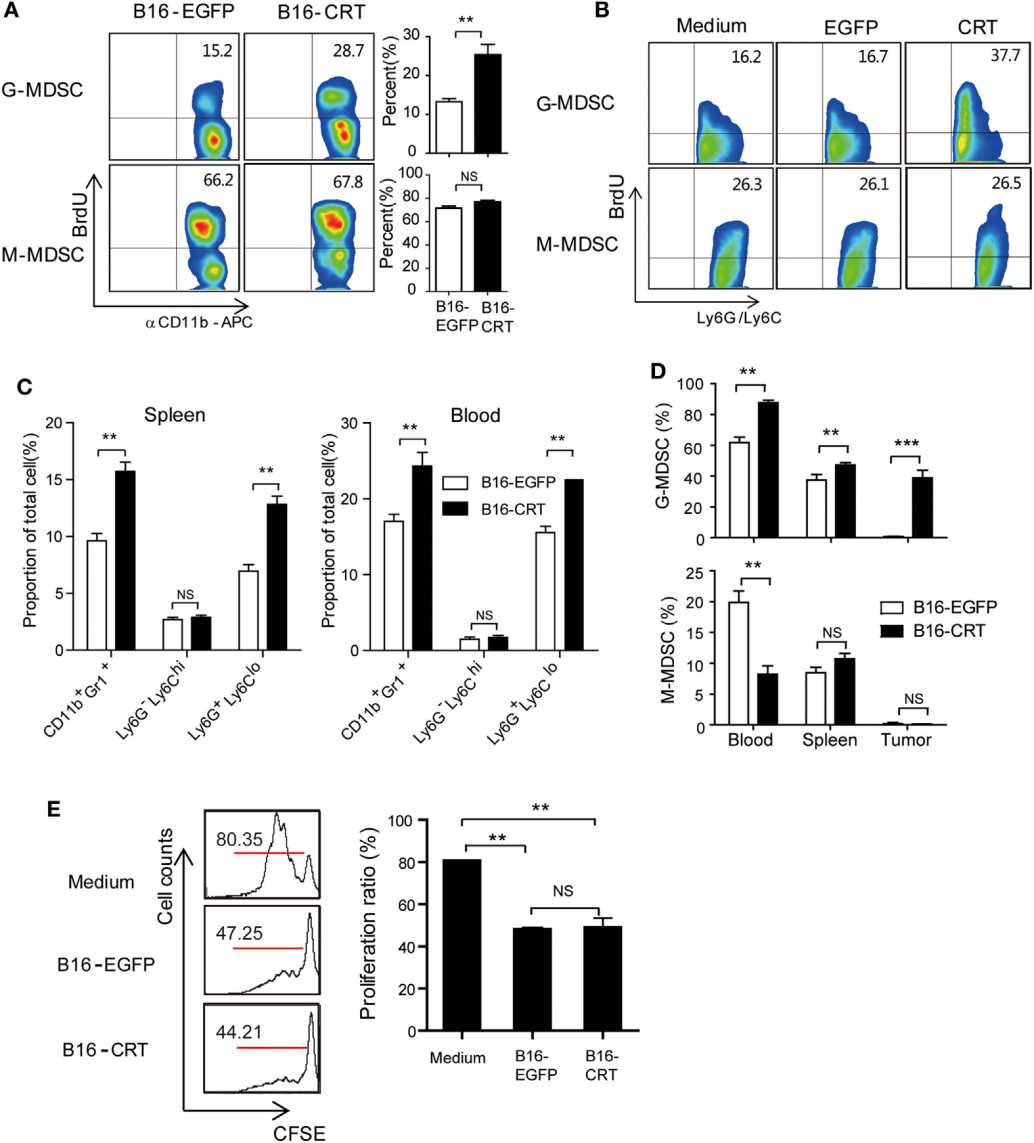
Increased myeloid derived suppressor cell (MDSC) accumulation in B16-CRT-bearing mice. **(A)** Groups of female C57BL/6 mice (*n* = 6) were s.c. injected with B16-EGFP or B16-CRT cells (5 × 10^6^/100 μl/mouse), followed by i.p. injection, on day 18, of BrdU (10 mg/ml in PBS, 200 μl/mouse). Twelve hours thereafter the mice were sacrificed for bone marrow cells. After staining with fluorescence-labeled Abs against BrdU, CD11b, Ly6C, or Ly6G, the cells were subjected to FACS analysis, gating on CD11b^+^Ly6G^hi^Ly6C^−^ (G-MDSC) or CD11b^+^Ly6G^lo^Ly6C^+^ (M-MDSC) populations. **(B)** For *in vitro* proliferation assay, bone marrow cells from C57BL/6 mice were cultured in the GM-CSF-conditional medium in presence of sCRT/39-272 or rEGFP for 3 days, followed by addition of BrdU to a final concentration of 10 µM directly to the culture for 2 h. Proliferation of MDSCs was analyzed by flow cytometry after staining with BrdU and fluorescence-labeled Abs against MDSC markers, gating on the CD11b^+^Ly6G^hi^Ly6C^−^ (G-MDSC) or CD11b^+^Ly6G^lo^Ly6C^+^ (M-MDSC) populations. **(C)** For assessment of MDSCs in the periphery, spleen and blood were collected from mice 21 days postinoculation of B16 tumors. Proportions of total MDSC (CD11b^+^Gr1^+^), G-MDSCs and M-MDSCs among splenocytes and WBCs were analyzed by FACS. **(D)** C57BL/6 mice (CD45.2)-bearing B16-EGFP or B16-CRT tumors (inoculated 17 or 10 days earlier, respectively) were adoptively transferred with CD45.1^+^ bone marrow cells (freshly isolated from CD45.1 transgenic mice), 5 days later MDSC percentages among CD45.1^+^ cells in the spleen, blood, and solid tumor tissues of the recipient mice were analyzed by FACS. **(E)** T cell proliferation inhibition assay. MDSCs isolated from B16-EGFP- or B16-CRT-bearing mice were incubated with 10^5^ Dye eFluorR 670 (eBioscience)-labeled splenocytes from OT-I mice at a ratio of 1:2 in the presence of OVA257–264 peptide (5 µg/ml) for 3 days. Representative FACS dot plots are shown in left panel and the statistic results on the right. The results were replicated for three times with consistent trend, representative results of the three repeating experiments are shown. ***p < 0.001, **p < 0.01. NS: not significant.

CD11b^+^ immature myeloid cells (IMCs), derived from hematopoietic stem cells in the bone marrow, are the source of common myeloid progenitor cells that differentiate into DCs (CD11b^+^CD11c^+^) or macrophages (CD11b^+^F4/80^+^) upon appropriate stimulation in peripheral organs (17). In the case of tumor-bearing individuals, however, IMCs accumulate in tumor microenvironment where they stop differentiation and functionally behave as MDSCs (17), which is accompanied by increased IMC percentage in the bone marrow as well as periphery (18). Based on our above shown results, we reasoned that tumor-derived sCRT39-272 might further enhance IMC/MDSC generation/proliferation in tumor-bearing animals. To assess this possibility, BrdU was i.p. injected into C57BL/6 mice-bearing B16-CRT or B16-EGFP solid tumors, 12 h later BrdU-positive cells among bone marrow CD11b^+^Ly6G^+^Ly6C^low^ G-MDSCs and CD11b^+^Ly6G^−^Ly6C^high^ M-MDSCs were numerated by flow cytometry. As shown in Figure 3A, in the G-MDSC population of bone marrow cells from B16-CRT-bearing mice, BrdU-positive cells accounted for 28.7%, significantly higher than that (15.2%) from the B16-EGFP controls, indicating accelerated proliferation of these T cell suppressing cells in the presence of sCRT-producing tumors. However, there was no significant difference in the proportion of BrdU-positive cells among bone marrow M-MDSC subset from mice-bearing B16-CRT or B16-EGFP tumors (66.2 vs. 67.8%). The selective expansion of G-MDSCs by rCRT was further confirmed by *in vitro* experiments, in which bone marrow cells were treated in GM-CSF-conditioned medium in the presence of sCRT/39-272 (Figure 3B).

Increased proliferation of G-MDSCs in the bone marrow of tumor-bearing mice was also reflected in the periphery. Compared with the B16-EGFP-bearing animals, B16-CRT-bearing mice showed a significant increase of G-MDSCs among splenocytes (13 vs. 7%) and WBCs (23 vs. 16%) (Figure 3C). Percentages of M-MDSCs among splenocytes and WBCs remained relatively low in both groups and difference between them was not statistically significant.

To exclude the possibility that the more significant generation of G-MDSCs in B16-CRT-bearing mice was purely due to the larger size of the solid tumors, C57BL/6 mice (CD45.2)-bearing B16-EGFP or B16-CRT tumors (inoculated 17 or 10 days earlier, respectively) of approximately the same size were adoptively transferred with CD45.1^+^ bone marrow cells (freshly isolated from CD45.1 transgenic mice), 5 days later MDSC percentage among CD45.1^+^ cells in the periphery of the recipient mice were monitored. Figure 3D shows that proportion of G-MDSCs in the CD45.1^+^ population in the spleen, blood, and solid tumors was significantly higher in B16-CRT-bearing mice than that in B16-EGFP controls. By contrast, percentage of M-MDSCs among the CD45.1^+^ population in the blood was significantly lower in B16-sCRT-bearing mice than that in B16-EGFP controls (7 vs. 19%), while there was no significant difference in the spleen and solid tumors. These results together suggest that the development of sCRT-secreting solid tumors is associated with more significantly increased proliferation/generation and output of G-MDSCs *in vivo*.

T lymphocytes are the main targets for MDSC suppression. To assess whether sCRT-generated MDSCs were functionally different from their conventional counterparts, MDSCs isolated from mice-bearing B16-CRT and B16-EGFP tumors were compared for ability to inhibit T cell proliferation *in vitro*, no significant difference was observed (Figure 3E).

### Recombinant sCRT39-272 Blocks G-MDSC Differentiation into DCs *In Vivo* and *In Vitro*

**Figure 4:**
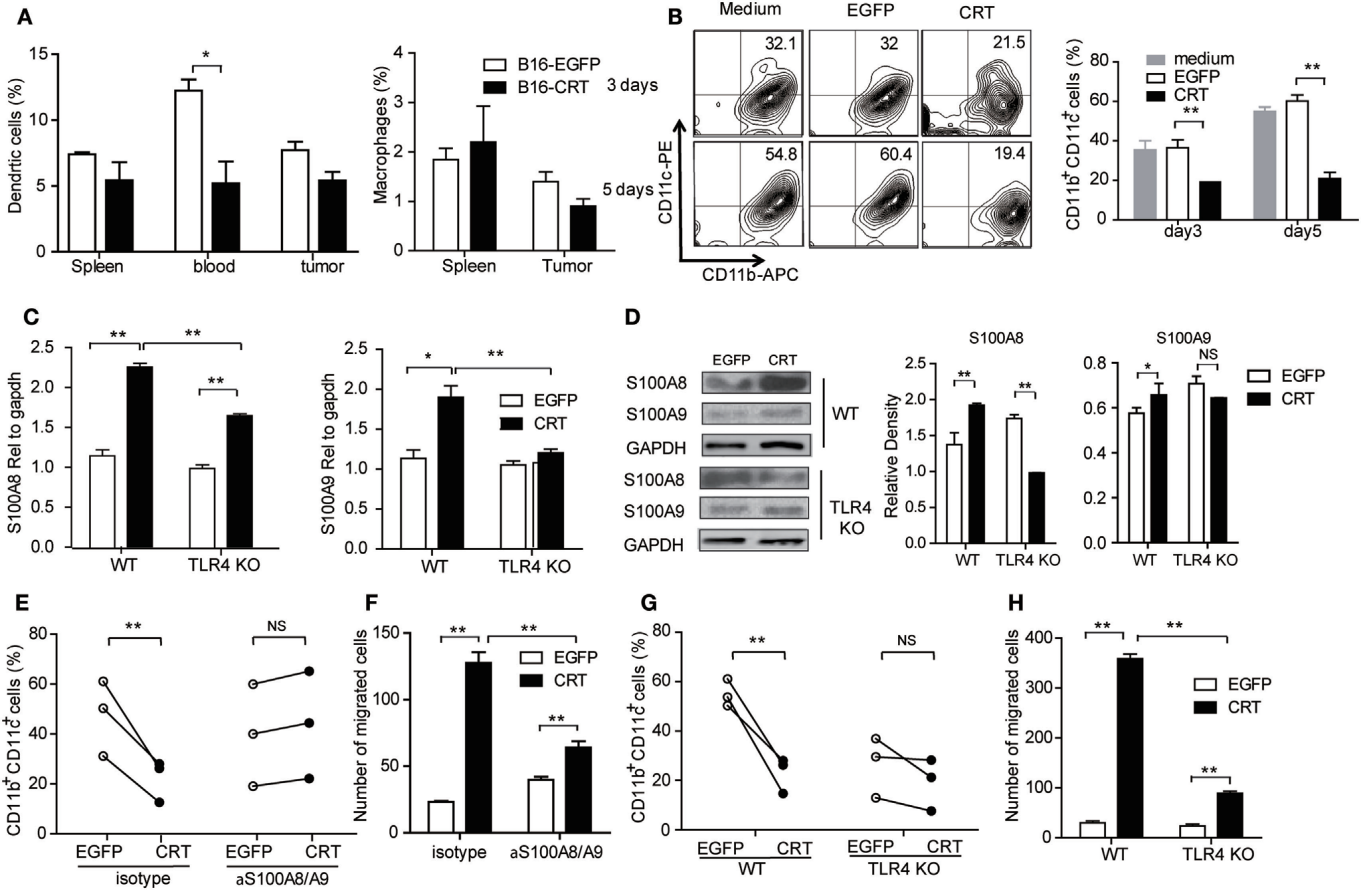
Calreticulin (CRT) mediates Gr1^+^ myeloid derived suppressor cell (G-MDSC) regulatory effect *via* TLR4/S100A. **(A)** Groups of female C57BL/6 mice (*n* = 6) were s.c. injected with B16-EGFP or B16-CRT cells (5 × 10^6^/100 μl/mouse) and sacrificed 21 days later for their spleens, blood, and solid tumors. Percentages of dendritic cells (DCs) (CD11b^+^CD11c^+^) and macrophages (CD11b^+^F4/80^+^) splenocytes, WBCs, and tumor cells were analyzed by FACS. **(B)** G-MDSCs purified from splenocytes of tumor-bearing mice were treated with GM-CSF (10 ng/ml) in the presence with sCRT/39-272 or, recombinant enhanced green fluorescence protein (rEGFP), for 3 and 5 days, followed by staining with fluorescence-labeled with anti-CD11b and anti-CD11c Abs and FACS analysis for double positive cells. **(C,D)** G-MDSCs purified from the spleens of tumor-bearing wide-type or TLR4-KO mice were treated with 1 µg/ml recombinant sCRT/39-272, or rEGFP, for 4–12 h, followed by RNA extraction and Q-PCR analysis for S100A8/A9 expression **(C)**, or immunoblotting assays using rabbit polyclonal Abs against S100A8 or S100A9, Abs against GAPDH were included as control **(D)**. **(E)** Purified MDSCs (2 × 10^5^) from spleens of tumor-bearing C57BL/6 mice were pretreated with anti-S100A8/A9 Abs (20 µg/ml) or 30 min, and then stimulated by GM-CSF in the presence of 1 µg/ml sCRT/39-272 or rEGFP for 5 days. The cells were then stained with fluorescence-labeled anti-CD11b and anti-CD11c Abs and analyzed by FACS. **(F)** Purified MDSCs (2 × 10^5^) from spleens of tumor-bearing mice were pretreated with anti-S100A8/A9 Abs (20 µg/ml) for 30 min in the top chamber of transwell. Recombinant sCRT/39-272 or rEGFP (1 µg/ml) was placed in the bottom chamber as chemotractant. After 3 h, transwell filters were fixed with 4% paraformaldehyde (PFA) in PBS, followed by DAPI staining and microscopic observation, average number of migrated cells in six different vision scope is shown. **(G,H)** G-MDSCs were purified from tumor-bearing WT or TLR4-KO mice were employed in studies for DC maturation and migration. The experiment was repeated for three times. **p < 0.01, *p < 0.05, NS, not significant.

Next, we asked whether B16-CRT tumors would be more powerful than B16-EGFP tumors in blocking MDSC differentiation into DCs or macrophages *in vivo*, which could be reflected by percentage changes in DCs and/or macrophages in the periphery. Figure 4A shows that percentage of DCs (but not macrophages) among WBCs of B16-CRT-bearing mice was significantly lower than that of B16-EGFP-bearing animals (5.5 vs. 12.5%). Decreased DC percentage in the spleen and tumor tissues was also observed in B16-CRT-bearing mice albeit not statistically significant. To further assess whether sCRT could directly suppress differentiation of MDSCs toward DCs, murine splenic G-MDSCs derived from tumor-bearing mice were incubated with GM-CSF for 5 days in the presence of recombinant sCRT39-272 or rEGFP followed by flow cytometric analysis for CD11b^+^CD11c^+^ DCs. Apparently sCRT39-272, but not rEGFP, substantially inhibited DC differentiation *in vitro* (Figure 4B).

S100A8 and S100A9 are proinflammatory proteins essential for functional maturation and chemotactic migration of MDSCs (19, 20). Interestingly, S100A8/9 mRNA expression level, quantitated by Q-PCR, was significantly higher in murine splenic G-MDSCs under stimulation with sCRT39-272 compared with rEGFP. This difference was partially (S100A8), or completely (S100A9), diminished when murine splenic G-MDSCs from TLR4-knockout mice were employed (Figure 4C), indicative of the involvement of TLR4 in sCRT-assisted G-MDSC generation which is in line with our previous observation that sCRT39-272 activates myeloid cells through interaction with CD14 and TLR4 (10). The same phenomenon was also observed on protein levels as evidenced by Western blotting assays on splenic MDSCs of tumor-bearing mice treated with sCRT39-272 or rEGFP (Figure 4D). Moreover, neutralizing antibodies against S100A8/9 effectively reversed sCRT39-272-mediated suppression of DC differentiation from G-MDSC as well as sCRT39-272 inducing G-MDSC migration *in vitro* (Figures 4E,F). Note that G-MDSCs from TLR4-knockout mice were poor responders to sCRT-induced differentiation toward DCs and migration *in vitro* (Figures 4G,H). Thus, sCRT39-272 could directly interact with MDSCs derived from tumor-bearing animals through TLR4, leading to production of S100A8/9, which in turn blocks MDSC differentiation. This at least partially explains the increased number of G-MDSCs in B16-CRT tumor-bearing mice.

### Increased Malignancy of B16-CRT Is Dependent on the Presence of Competent MDSCs

**Figure 5:**
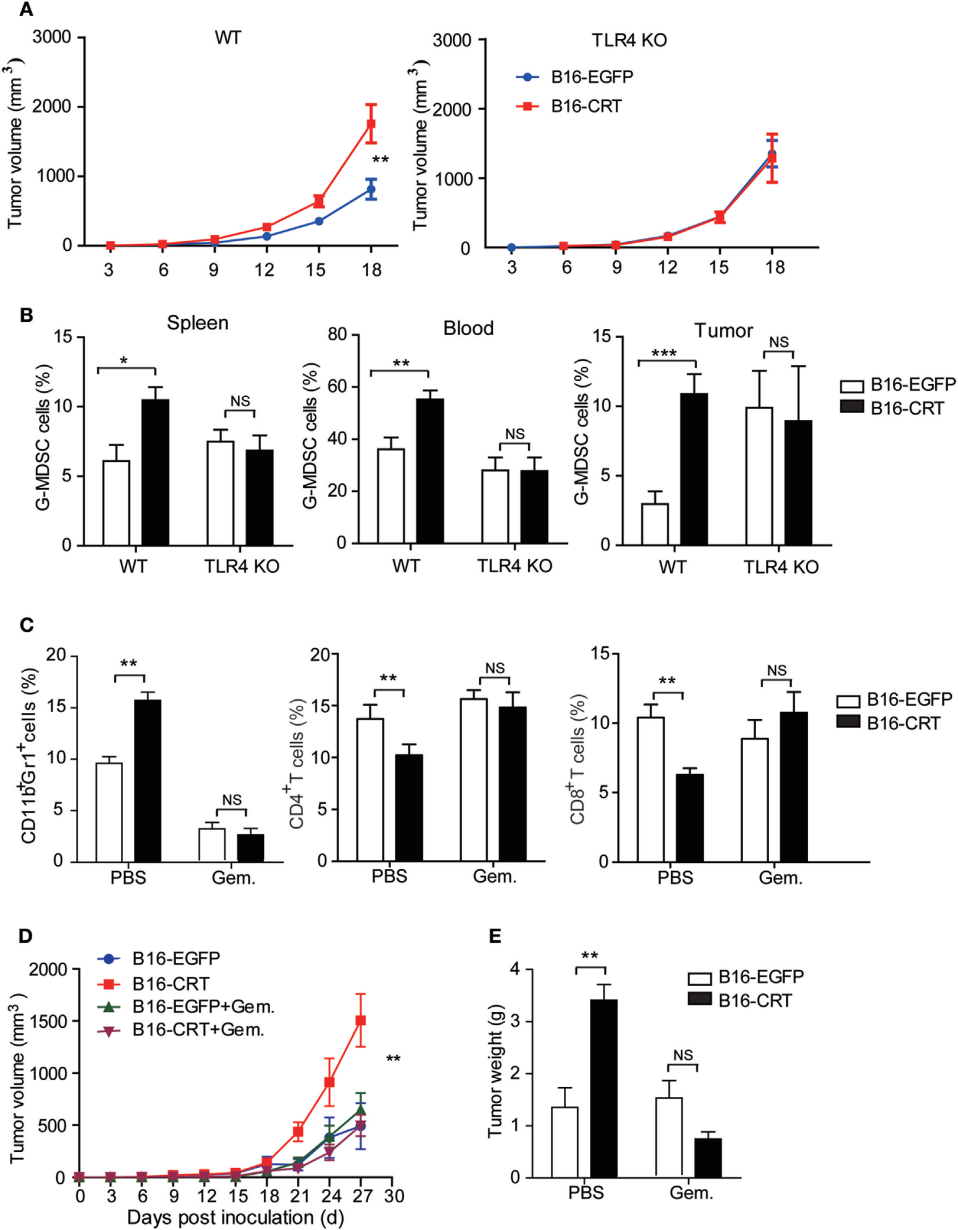
Malignancy promoting effect of sCRT39-272 is dependent on the presence of myeloid derived suppressor cells (MDSCs) *in vivo*. **(A)** Groups of female C57BL/6 or TLR4-KO C57BL/10 mice (*n* = 6) were s.c. injected with B16-EGFP or B16-CRT cells (5 × 10^6^/100 μl/mouse), followed by measurement of solid tumors formed at the sites of injection every 3 days. **(B)** The mice were sacrificed on day 18 for their spleens, blood, and solid tumors. Percentages of Gr1^+^ myeloid derived suppressor cells (G-MDSCs) (Ly6G^+^CD11b^+^) among the splenocytes, WBCs, and tumor cells were analyzed by FACS. **(C–E)** Groups of female C57BL/6 mice (*n* = 6) were s.c. injected with B16-EGFP, or B16-CRT, cells (5 × 10^6^/100 μl/mouse), followed by gemcitabine (100 mg/kg, i.p.) or vehicle treatment every 3 days until their sacrifice on Day 27. Then cells from the spleens, blood, and solid tumors were stained for CD11b, Gr1, CD4, and CD8 for flow cytometric analysis **(C)**. Tumor volume was measured every 3 days and tumor growth curve shown in panel **(D)**. Tumor weight measurement results are given in panel **(E)**. The experiments were repeated for three times. *p < 0.05, **p < 0.01, ***p < 0.001. NS, not significant.

Data accumulated thus far indicate that the malignancy-enhancing effect of sCRT/39-272 is related to TLR4^+^ MDSCs *in vivo*. Indeed, when TLR4-knockout mice were employed as recipients to B16-CRT and B16-EGFP tumors, no significant differences in tumor size, G-MDSC percentage in the blood and spleen, and infiltrating MDSCs in solid tumor tissues were observed between the two groups (Figures 5A,B). Taking this further, gemcitabine was utilized to selectively deplete MDSCs in tumor-bearing C57BL/6 mice (21). The depletion efficacy was confirmed by significant reduction of CD11b^+^Gr1^+^ MDSCs in tumor-bearing mice given gemcitabine injections (Figure 5C). In the MDSC-depleted animals, no significant difference was observed between the B16-CRT and B16-EGFP groups in terms of solid tumor growth (volume and weight) up to 27 days after inoculation (Figures 5D,E), strongly supporting our hypothesis that MDSCs are essentially important for the enhanced malignancy of B16-CRT tumor cells. It is also of interest to note that gemcitabine treatment reversed the suppression of CD4^+^ and CD8^+^ T cell proportions in B16-CRT-bearing animals in these experiments (Figure 5C).

## Discussion

Several studies have shown that CRT and its fragments exhibit anti-tumor effects in animal models (6, 16, 22), which is attributed to the so-called vasostatin activity of CRT N domain (1–180) (6) and other immunobiological roles of CRT (7–9). In our study, however, sCRT39-272 did not suppress the growth of blood vessel endothelial cells *in vivo* as there were more vascular tubes in the B16-CRT than the B16-EGFP solid tumors (not shown), arguing against an antiangiogenesis effect for CRT. Evidence presented here clearly suggests a malignancy promotion role for sCRT mainly through TLR4- and S100A8/9-mediated MDSCs differentiation/generation and recruitment. Liu and colleagues have reported that the vasostatin N-domain of CRT caused enhanced malignant behavior of neuroendocrine tumor cell line, BON *in vivo* (23). The mechanisms include enhancing adhesion and invasion of BON cells through down-regulating tumor suppressor genes including p53, nm23, Rb and vinculin. It should be noted that, in some of the studies on anti-tumor effect of CRT, immunodeficient mice were employed (16, 22). In such cases, the influence of MDSCs in tumor development would be missed.

Chronic inflammation microenvironment is known to be of great importance to MDSC accumulation (24) and tumor development (25). Tumor-derived sCRT39-272 should be able to help sustaining such an inflammatory microenvironment because of its potent ability to elicit proinflammatory cytokine production by monocytes/macrophages *via* CD14/TLR4 as documented by our previous work (14, 15). We have also previously reported that sCRT39-272 could induce regulatory B cells *in vivo*, which could ameliorate experimental allergic encepharolitis in mice (26). Anti-CRT IgG Abs, induced by sCRT39-272 immunization, showed a regulatory effect toward T cell-mediated cellular immunity (27). Whether these immunoregulatory factors could also contribute to enhanced B16-CRT malignancy remains to be investigated.

Cell surface-bound CRT (ecto-CRT) and secreted sCRT may play opposite roles in tumor development. Ecto-CRT on tumor cell surface has been shown to be an important ligand for recognition and attack by phagocytic cells (8). In contrast, free CRT molecules released by tumor cells mainly function as a TLR4 agonist against myeloid cells, leading to inflammatory cytokine production (14, 15). When tumor cells die of apoptosis (as a result of chemotherapy, irradiation or hypoxia), CRT is likely to translocate to the membrane surface and can function as an “eat me” signal. In the case of necrotic cell death, however, CRT is more likely to be released in soluble form that may contribute to a chronic inflammatory niche as well as MDSC generation/accumulation necessary for tumor development.

**Figure 6:**
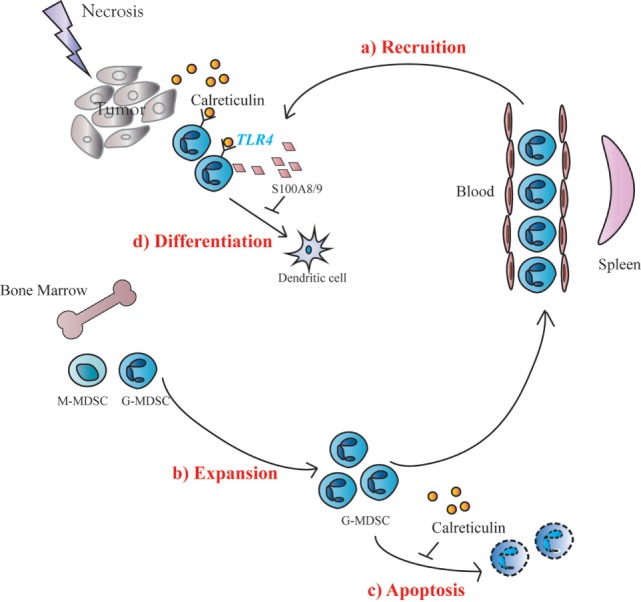
Schematic representation of soluble calreticulin (sCRT) activity on tumorigenesis. When sCRT or its fragment was released from tumors under pressure, sCRT showed a protumorigenesis activity, because of accumulation of Gr1^+^ myeloid derived suppressor cells (G-MDSCs) *in vivo*. The possible molecular mechanisms include: (a) sCRT could recruit MDSCs in tumor sites from blood; (b) IMC in bone marrow expanding when stimulated by sCRT; (c) the apoptosis of MDSCs could also be inhibited by sCRT; and (d) differentiation of MDSCs toward DC could be inhibited by sCRT.

Generation and survival of MDSCs depends on the presence of growth factors including M-CSF, GM-CSF and G-CSF as well as proinflammatory cytokines. However, mechanism for the conversion of IMCs to MDSCs is even more complex (10). An important finding of the present study is that sCRT39-272 could act directly upon IMCs *via* TLR4, leading to (i) increased expression of S100A8/9; (ii) reduced maturation/differentiation into DCs or macrophages; (iii) selective MDSC chemotactaxis; and (iv) apoptosis inhibition, all of which help accumulation of MDSCs in the tumor microenvironment (Figure 6). The important roles of S100A8/9 in chronic inflammation, MDSC generation and recruitment have also been documented by previous groups (19, 20). CRT can be regarded as a damage-associated molecular pattern (DAMP) or heat shock protein (HSP) that are able to trigger inflammatory responses of innate immune cells by engaging TLRs (28). Interestingly, many DAMPs (e.g., S100A) and HSPs could also engage TLRs to promote accumulation of MDSCs (24, 29, 30). In addition, inflammatory cytokines including IL-6, TNFα could also be induced by sCRT (13), which could in turn drive the generation of MDSCs (19, 31). Taken together, results presented herein suggest that CRT is a double-edged sword in tumor development. On one hand, it is recognized by the immune system as a major TAA. On the other, it facilitates MDSC accumulation in tumor tissues through various routes.

## Ethics Statement

This study was carried out in accordance with the recommendations of Utilization Committee of Soochow University. All research protocols were approved by the Soochow University of the Animal Care and Utilization Committee and approved by the local government authorities.

## Author Contributions

F-YG designed the research. X-YH, YC, F-YG, ZZ, and ZG carried out the experiments. X-YH, YC, and F-YG analyzed the data. F-YG and X-MG prepared the manuscript. All authors discussed the results and commented on the manuscript.

## Conflict of Interest Statement

The authors declare that the research was conducted in the absence of any commercial or financial relationships that could be construed as a potential conflict of interest.
